# Towards a Healthcare Innovation Scaling Framework—The Voice of the Innovator

**DOI:** 10.3390/ijerph192315515

**Published:** 2022-11-23

**Authors:** Chipo Nancy Ngongoni, William Wasswa, Lindiwe Makubalo, Matshidiso Moeti, Moredreck Chibi

**Affiliations:** World Health Organization African Region, Brazzaville P.O. Box 06, Congo

**Keywords:** innovation intermediary, innovation ecosystem, innovators, ecosystem management, healthcare

## Abstract

This paper investigates the systemic challenges that African healthcare innovators experience in the quest to scale their innovations. The aim is to aggregate insights and to conceptualize a foundation towards building a framework that can be used as a guide by intermediary organizations and global partners to support collaborative innovation in African countries. These insights were gained from analyzing a dataset of survey responses obtained from a follow-up on 230 innovators who took part in the inaugural WHO Africa Innovation Challenge that was held in 2018. The insights led to the identification of 10 key foundational blocks that assist in ecosystem management in a bid to strengthen national health innovation ecosystems and to improve the sustainability and integration of innovations in the health system.

## 1. Introduction

One of the cornerstones for achieving Universal Health Coverage (UHC) and the health-related Sustainable Development Goals (SDGs) is to ensure affordable access to quality essential medicines, health products and services [1]. Globally, the current drive to achieve UHC has been characterized by the proliferation of innovative interventions aimed at enhancing life expectancy, quality of life, and diagnostic and treatment options, as well as efficiency and cost effectiveness of the healthcare system [2,3].

Africa has a unique opportunity to leverage medical innovations and to invest in new healthcare delivery models to close the healthcare gap. This is evident as the demand for innovations across the continent continues to increase due to emerging socio-economic dynamics, for instance, a burgeoning youth population; unplanned rural–urban migration; epidemiological changes including non-communicable diseases; and climate change. The growing body of evidence shows that the future of health in Sub-Saharan Africa will be underpinned by the development and adoption of home-grown innovations adapted to a country’s specific needs [4]. In the last 15 years, over USD 12 billion dollars of investment has been channeled towards the development of health interventions to address major global health challenges such as HIV/AIDS, Tuberculosis and Malaria [5]. This has seen a surge in health innovations across the continent. However, the biggest barrier has been a lack of a systematic way of harnessing and scaling up these innovations to meet the local needs of the African people.

Historically, the development of innovations has been concentrated in high-income economies to meet their demand. However, there is a notable shift in the center of gravity to the innovation landscape where developing countries, particularly in Africa, could ‘leapfrog’ their current health systems by leveraging off innovations due to lower sunk costs related to existing infrastructure and equipment, rapidly increasing technology penetration, alternative operating and financing models, and possibly less structured regulatory environments [6]. As a result, new innovative health solutions can be deployed quickly and with immediate impact without the need to proportionately increase healthcare facilities and professionals.

As the deadline for the 2030 agenda for Sustainable Development is fast approaching, the data from the 2019 Sustainable Development Goals (SDGs) report shows major progress in improving the health of millions of people [7]. Maternal and child mortality rates have been reduced, life expectancy continues to increase globally, and the fight against some infectious diseases has made steady progress. Despite progress in other parts of the world, Africa’s health indicators remain behind those of other continents, and major health inequities exist. Health outcomes are even worse in fragile countries, rural areas, urban slums, and conflict zones. Conditions are even more dire among people who are poor, disabled, or marginalized. Therefore, a systemic framework shift is needed to deliver better health outcomes in African countries through people-cantered health systems approach. For instance, huge scope and potential exists for innovative and low-cost new vaccines, diagnostics, therapies, and information technology applications for prevention and care [4].

Due to the importance of contextually relevant interventions in the improvement of health delivery services, there is growing acknowledgment of the need for locally driven innovative solutions and to make the best use of scarce resources [8]. Therefore, it is important for countries in Africa to chart their own sustainable path for innovation to improve health outcomes, while making maximal use of international experiences and evidence, strengthened stewardship of health, and commitment to accountability. To deliver on this potential, countries require an innovation ecosystem that rigorously identifies health priority needs, clearly defines health problems to be solved, and effectively delivers at scale affordable and appropriately designed innovative health solutions in an agile fashion.

## 2. Related Work

The healthcare innovation ecosystem involves various actors that interdependently co-create value through their interconnections and interlinkages to achieve sustainable healthcare improvements through innovation and technology development [9]. These actors often include governments, private sector firms, universities and other public research institutions, individual innovators and entrepreneurs. Recognizing the complex and dynamic nature of the innovation ecosystem, it is important to create a system through alignment of actors to drive innovation outputs for sustainable impact. Exemplified below are some examples of how innovation ecosystem dynamics affect the scaling of innovations.

### 2.1. Mobile Alliance for Maternal Action (MAMA) and MomConnect

The Mobile Alliance of Maternal Action (MAMA) digital platform was launched in South Africa in 2011 to help address the information gap for pregnant and post-natal women in order to reduce maternal and infant mortality. The program was a Public Private Partnership involving the United State Agency for International Development (USAID, Washington, DC, USA), Johnson & Johnson, United Nations Foundation (New Brunswick, NJ, USA) with the implementing Praekelt Foundation (Johannesburg, South Africa), Cell-Life (Providence, RI, USA), Always Active Technologies and Vodacom (Durban, South Africa) [10]. The platform was available across five channels, namely voice, SMS, Unstructured Supplementary Service Data (USSD), mobile websites and Mxit (a South African mobile phone-based chat platform that later closed in 2015) [10]. Over the course of three years, the number of subscribers rose to over 500,000, but this was relatively a low growth compared to what had been planned and projected [10,11]. The main disparity in the MAMA platform that affected the growth of the platform had been that the government was not involve in the inception of the project. Since the MAMA partners had carried out a lot of groundwork, the project was handed over in 2013 to the South African National Department of Health, who became the primary stakeholder of the platform.

The platform was renamed MomConnect, where it ended up with over 20 partners with some implementing partners still in the team. The channels were initially reduced to just USSD and SMS, and the signup process was not confusing and was now through the clinics and community health workers, which opened a wider reach for the MomConnect platform. To date, the platform has connected over two million pregnant women and new mothers to vital services and information in South Africa whilst also having implementations in other countries such as Nigeria [12]. Though MAMA was superior in terms of technology, the success of MomConnect has been attributed to government stewardship, user centric designs, clear technology frameworks [13] and better stakeholder management [14]. Interestingly, MomConnect gave rise to extensions in the form of NurseConnect (a platform for nurses to share information), ChildConnect (a platform to support and educate parents and caregivers through an SMS curriculum of ECD content) and HealthConnect (a platform to disseminate COVID-19 verified information from the NDOH). The morphing of MAMA to MomConnect is one of many cases where scaling is affected by ecosystem dynamics, which is why ecosystem dynamics are important to address.

### 2.2. District Health Information System 2 (DHIS2)

Another example of a health innovation that scaled with different ecosystem dynamics is the District Health Information System 2 (DHIS2). This is an open-source mobile and web-based platform used for data collection and as a health management information system (HMIS). DHIS2 comes with purportedly easy-to-interpret analytics using customized charts, pivot tables, maps and dashboards. It has a web-based portal that facilitates translation into several local languages. DHIS2 has been utilized in the delivery of various service delivery interventions, and in most countries, the expert knowledge, skills and capabilities for maintaining the platform usually lie outside the health ministries’ organizational boundaries.

DHIS2 started as a doctoral project by Jørn Braa in 1997 through a collaboration between the University of Cape Town and the University of Oslo (UiO) under the Health Information Systems Program (HISP) [15]. The aim of the doctoral study was to decrease the inequality that had been created in the public health sector due to the apartheid era. Professor Jørn Braa has led the growth of the project through a network of over 17 in country and regional HISP programs and action research through doctoral and masters students to be deployed in over 73 low and middle-income countries and more than 100 countries worldwide [16]. The core pillar of the platform’s success was based on governments taking ownership of the installed DHIS2 instances and creating in-country capacity. This has helped build an organic innovation ecosystem consisting of a global community of monitoring and evaluation specialists, health professionals, software developers and such as well as an expert partner network that is part of a community of practice [17]. Moreover, sustainability and impact issues are at the core of the platform, which has seen a change in the way that the capacity building training academies are undertaken. Firstly, the annual training conference changed from being a training course to being a forum for the community to share best practices and lessons learnt whilst implementing their instances of DHIS2. This saw the training and capacity building becoming decentralized to regional academies and the fundamentals made readily available online.

### 2.3. Aim of the Study

These cases were exemplified to cement the reason why we undertook the study to try to see what the constraints can be to scaling innovations that innovators face and how those can be addressed. Hence, the purpose of this paper is therefore threefold. Firstly, to participate in the discourse around the identification of what key aspects are important for innovators when supporting the scaling of innovations or promoting the use of local innovations in Africa. Secondly, the identification of key cornerstone factors that are important in the management of innovation ecosystems, with emphasis on an empirically informed perspective of indicators that can be utilized in the analysis of innovation ecosystems and the ecosystem actors’ competences and capabilities. This is a key aspect that is still nascent in innovation ecosystems’ discourse and research [18,19,20]. Thirdly, the paper seeks to develop an innovation ecosystem framework that is informed by the needs of innovators from Africa to facilitate the end-to-end process for innovation development until sustainable scaling up. The framework will serve as a reference tool highlighting key areas and attributes that need attention in strengthening the local health innovation ecosystem. Guidance tools that assist innovation intermediaries or key stakeholders are fundamental ways to assist in ensuring the creation of an environment that is conducive for continuous innovation. Hence, the study stems from addressing the question of ‘*What are the key aspects that innovation intermediaries need to look at in order to effectively assist in the value co-creation process for actors in the healthcare innovation ecosystem*?’ This paper takes the position that understanding the evolution of an innovation ecosystem from the perspective of innovators including understanding enablers or factors that deter progress aids in ensuring sustainability of the ecosystem.

The paper progresses as follows: the next section gives an outline of the methodology that was utilized in this study and how the dataset that is utilized in this study was collected. The results of the survey are outlined, highlighting some systemic challenges in the innovation ecosystem. This is followed by the identification of the key leverage points, which outline the pillars of the framework will be discussed, and then, conclusions, implications and limitations of the study will also be put forward. This is with emphasis on the critical role that an enabling environment plays in scaling innovations.

## 3. Materials and Methods

This study utilized a longitudinal approach based on a prior study. The first study was conducted in 2020 as a follow-up study on the top 30 innovators that had been selected from the inaugural 2018 World Health Organization Regional Office for Africa (WHO AFRO) Innovation challenge [21]. The innovation challenge was a global call for innovators, including youth and women, to submit their innovative and novel solutions aimed at addressing unmet health needs across Africa. The challenge sourced innovations under three broad categories:

- Product or technological innovations that contribute to the research, development and design of new products or improvements in existing products;
- Process or service innovations such as innovative financing mechanisms;
- Social innovations.

The solution requirements were that it is relevant to Africa, innovative and scalable. The aim of the first study was to assess the progress that the innovators had made towards scaling their innovations. Data for that preliminary study were collected through a survey, and the observations made after the 2-year follow up of the 30 innovators provided the impetus for additional follow-up on all the 2415 participants of the Innovation Challenge. Eighteen of the innovators had made quantifiable progress in scaling up their innovations, while twelve had abandoned their projects due to various reasons. This was a sizeable 40% who has stalled in their progress, and the innovators cited several key barriers to scaling, most of which were beyond the health sector.

**Figure 1:**
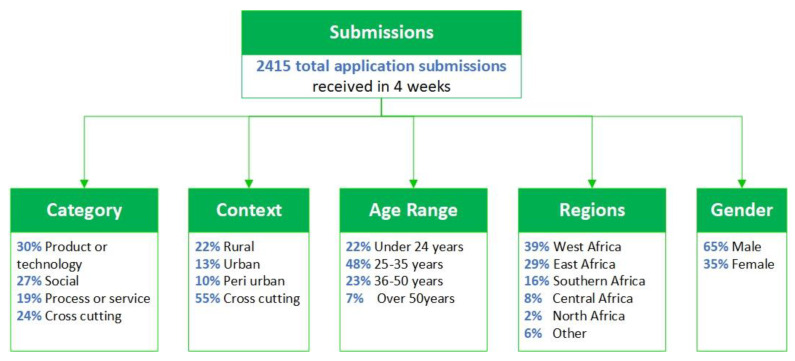
Innovator profiles from the 2018 Innovation Challenge.

Hence, for this study, a survey was sent out to all the previous participants (*n* = 2415) of the inaugural edition of the WHO Africa Innovation Challenge in 2018 [22].The profiles of the innovators that took part in this challenge are shown in Figure 1 below.

The survey consisted of various questions aligned with the progress that the innovators had made over the course of four years since the launch of the Innovation Challenge in 2018. There were 230 respondents, with a 10% completion rate. The survey responses were collected using SurveyMonkey developed by Zendesk (California, CA, USA) and the data were extrapolated in a csv file and later analyzed in Microsoft Excel developed by the Microsoft Corporation (Redmond, WA, USA).

**Figure 2:**
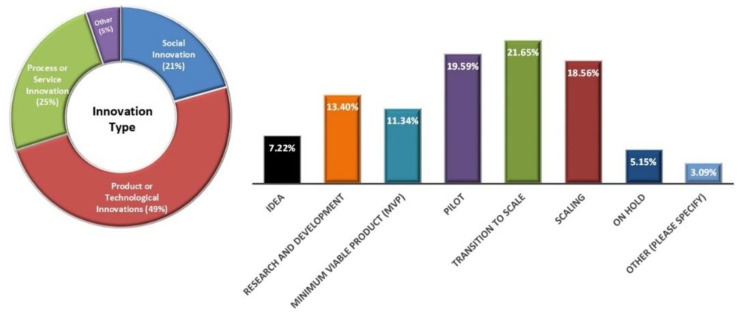
Survey responses as innovation descriptions and development stages.

The stages of innovation shown in Figure 2 ranged from being an idea, undergoing research and development, being developed into a minimum viable product, piloting or scaling to being on hold for various reasons.

## 4. Results

### 4.1. Survey Results

Two particular questions in the survey were dedicated to understanding the challenges that the innovators faced in scaling up their innovations and in gaining insights into their primary needs from the innovation ecosystem. These questions sought to generate insights on the challenges that the innovators were facing that was hindering their progress in various ways. Highlighting these areas helps articulate the key areas that the ecosystem builders or orchestrators need to be aware of in order to address the specific needs and support that the innovators expect from the ecosystem.

The questions were fielded as follows:

“*What are some of the key challenges that you are currently facing in executing or scaling up your innovation*? Please select all that are applicable to you.” The responses to that question are shown as a percentage of all the respondents in Table 1.

The other key question was “*What are the opportunities and support that you would want from key stakeholders in the healthcare innovation ecosystem? Please select all that are applicable to you*.” The results of that question are shown as a percentage of all the respondents in Table 2.

These two questions served two key purposes. One aim was to trace the progress that the innovators had achieved and to identify what that was. Notably, 88.66% of the innovators highlighted financial constraints as a key challenge that they face, 45.36% selected insufficient human resource capacity and 42.27% noted that a lack of standardized technology infrastructure in the healthcare sector affected their scaling. This could be due to 49% of the respondents working on a product or technological innovation. These issues are not new to entrepreneurial ecosystems.

Hence, the other importance of the second question considered in this study was to inform how key ecosystem intermediaries including the ministries of health can assist in the value creation process of innovators in a healthcare innovation ecosystem. Correlating their expectations with the needs from the innovation ecosystem, the top responses aligned with what the innovators need from the ecosystem: 93.81% identified funding, 65.98% requested linking to markets for their innovations, 62.89% wanted capacity building and 58.76% identified formal network building through open innovation platforms as important.

The responses of these two questions mentioned above were now mapped to see what core thematic areas came up from the analysis, which resulted in the identification of the 10 foundational aspects that formed the basis of the innovation scaling framework. The framework outlined leverage points that assist in strengthening the context specific innovation ecosystems across the entire value chain.

### 4.2. Towards a Healthcare Innovation Scaling Framework

**Figure 3:**
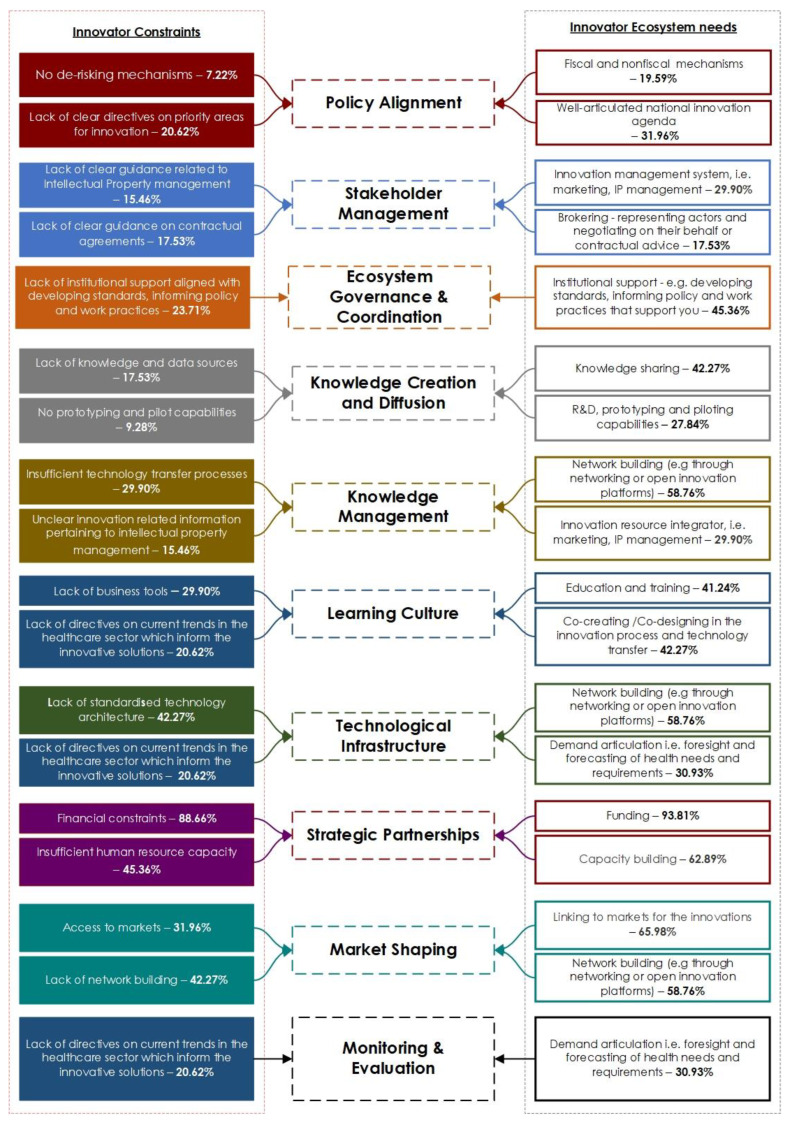
Ecosystem framework foundational pillars. These pillars were derived from the survey responses as key recurring themes and what they mean for the ecosystem.

This study attempted to formulate the Healthcare Innovation Scaling framework based on the themes that stemmed from the concerns and aspirational needs of the innovators in the healthcare innovation ecosystem. This led to the categorization of the 10 key facets that ecosystem actors and key stakeholders should take note of. These 10 facets are depicted in Figure 3 and described below:

1. *Policy alignment*: The government needs to develop a set of interventions including measures, programs, incentives, and other instruments aimed at supporting the creation and diffusion of innovations. These include the interaction, articulation and coordination of a set of policies within a complex environment with the purpose of achieving specific and desired innovation outcomes. This is shown through a proactive and supportive government that develops and implements effective policies and incentive mechanisms [8]. It is key to address policy disparities through promoting collaboration by building trust, policy sharing and co-design across health systems, which breaks down silos between governments and agencies and innovators [23].
2. *Stakeholder management* entails the facilitation of synergies and interconnectedness through value co-creation amongst ecosystem actors. In healthcare, this is usually around public and private sector engagement mechanisms that promote trust building and articulate various actor roles and responsibilities. These processes entail identifying, analyzing, planning, executing and monitoring stakeholders using various techniques and tools [24]. Therefore, agile innovation ecosystems are a result of proactive stakeholder management, which assists in an alignment structure of the stakeholders, a group of partners, that are required to interact so that an important proposal can be implemented [25]. Managing stakeholders strategically is of importance to their sustainability.
3. *Ecosystem governance*: A proactive presence of high-level leadership to coordinate ecosystem activities is very important. Such proactiveness can be demonstrated through the mandates of forward-thinking strategies such as the WHO AFRO regional strategy [8]. The strategy encourages African member states to set up governance and management mechanisms for ensuring equitable and inclusive innovation [8]. Some key activities aligned with ecosystem governance are around innovation agenda setting, assessing institutional capacity as well as defining the core functionality of the ecosystem to ensure that a bottom-up approach to innovation is central to strengthening the innovation enabling environments for health systems. This enables representation, consultation and decision-making from different parts of the ecosystem which can speak to concerns from various ecosystem actors and can address power imbalances [26].
4. *Knowledge creation and diffusion*: This aspect entails having a clear strategy of how knowledge is generated and diffused across the entire innovation ecosystem. This is an important aspect which is the engine of innovation. Knowledge is a crucial economic resource and a source of lasting competitive advantage for any system [27,28]. The types of knowledge products in the ecosystem can be tacit or explicit, where explicit knowledge is formal or systematized and tacit knowledge is highly personal though interactions and cannot be formalized [29].
5. *Knowledge management*: This is the strategy for management of intellectual property to incentivize innovation development. It is not enough that knowledge is created, but true value comes from the management of this knowledge with support mechanisms. Important capabilities lie around the creation, acquisition, sharing and utilization of knowledge [30,31]. Managing knowledge effectively includes considering the absorptive capacity of the innovation ecosystem in integrating new, seemingly disruptive technologies into the healthcare innovation ecosystem across all facets such as manufacturing, supply chain management, facility management and local manufacturing of medical products.
6. *Learning culture*: Assessing the learning activities in the ecosystem and how to ensure continuous learning is important. Creating a learning health innovation ecosystem is essential in ensuring that the ecosystem evolves in an agile manner [32]. Such a culture is cultivated through the creation of an environment that promotes continuous engagement and new ways of thinking between innovators and key stakeholders that facilitate relationship building, ongoing dialogue and learning. Through these interactive engagements, policy makers are enlightened on the barriers that affect innovators in scaling their innovations in the health system, and on the other hand, innovators become enlightened on the strategic direction that the government is taking and incentives aligned with supporting their innovations. Such interactions not only support absorptive capacity of the innovations but also help different ecosystem actors connect to define problems and to create solutions themselves. With clarity early on and ongoing engagement, innovations can adapt and grow in directions that meet concrete development demands and can more easily tap partnerships and resources for scaling at later stages.
7. *Technological infrastructure*: The development of a technological base that supports development of innovations is very important. This entails identifying and clarifying standard architecture that is utilized across the core health systems and the policies around enabling such technologies, e.g., Information and Communication Technologies. Hence, collaboration with other policy makers is important in ensuring that standards are also inclusive and conscious of the infrastructural constraints in various contexts. Key considerations such as the interoperability of systems and visibility of the various technologies that are utilized across healthcare systems is important to be able to plan and include policy in the strategic initiatives across the ecosystem [33]. Inadvertently, this enables innovators to invest and develop context relevant innovations that can integrate into the health system.
8. *Monitoring and Evaluation*: The monitoring and evaluating (M&E) of innovation ecosystems and platforms is indeed a big challenging task due to the complexity. Hence, effectively monitoring is critical to ensure that health innovation ecosystems function effectively and to achieve their intended purposes. Monitoring aims to assess the functioning and effectiveness of integrated innovations to improve policy and practice, to develop capacity and to improve links among actors. The information that is gathered through the M&E process can be used to improve the management of the ecosystem, to change policies and to promote larger-scale changes. M&E is a key facet in a learning health system that seeks to document and value these changes. Monitoring is carried out around assessing activities, process outputs and outcomes and the results of the impact on the target beneficiaries.
9. *Strategic partnerships*: The 17th Sustainable Development Goal identifies how multi-stakeholder partnerships should be enhanced for sustainable development, and help mobilize and share knowledge, expertise, technology and financial resources. With the increasing rate of globalization, innovation has become a key differentiating transformative feature that defines long-term sustainable impact around innovative activities. The government is at the core of providing de-risking mechanisms for innovators whilst attracting investment by external partners to support the integration of local innovation into the health system. This is through effective public, public-private and civil society partnerships.
10. *Market shaping*: This should be an effort by the government to create a market for local innovations. An agile health system is inextricably linked to the health of the marketplace that delivers life-saving health products to low-income populations. Market shaping can disrupt current practices or transform existing market structures through creating efficiencies that lead to better health outcomes for the poor. Governments, donors and procurers can use their purchasing power, financing, influence, and access to technical expertise and policy shaping to address the root causes of market shortcomings and influence markets for improved health outcomes. When governments intervene in market shaping, the aim is to reduce long-term demand and supply imbalances and to reach a sustainable equilibrium where local producers and innovators are integrated into the health care supply chain. Market shaping provides the much-needed impetus for innovators to develop innovations that are linked to government demand.

## 5. Discussion

### Innovation Scaling Ecosystem Framework Possible Indicators

A framework without examples of possible indicators is futile as an instrument. Leendertse et al. [19] outlined how to measure what is happening in innovation ecosystems by looking at 10 aspects. These are formal institutions, entrepreneurship culture, networks, physical infrastructure, finance, leadership, talent, new knowledge, demand and intermediate services. There are some similarities with this study though our proposed framework goes further by incorporating feedback from innovators who are central in developing and scaling up innovations. Another innovation ecosystem framework that was proposed by Mulas et al. [34] outlined the pillars comprising people, infrastructure, economic assets, enabling environment and networking assets as key to boosting the innovation ecosystem in cities. This framework goes a bit further in not isolating usage of the framework to a specific region. The proposed framework in this paper also goes to a granular level and adds the importance of clear strategic alignment, ecosystem governance and stakeholder management.

The Deutsche Gesellschaft für Internationale Zusammenarbeit GmbH (GIZ) entrepreneurial ecosystem strengthening guide [35] and the Aspen Institute’s entrepreneurial diagnostic toolkits [36] opt to look from the perspective of accountability through quantitative indicators. These include aspects such as the number of entrepreneurial-centered programs, survival rate of the start-ups, incubators, number of meeting hours between ecosystem actors or matchmaking events. This metrics help in terms of quantifying the infrastructure and activity around the innovators. The indicators proposed in the framework in this paper stem from insights gained from the innovators and what hampers their progress in terms of scaling their innovations. These categories are outlined in Table 3.

The suggested indicators for innovation ecosystems are such that there is some mechanism present that helps track and trace the actor interactions and activities in the innovation ecosystem. Drawing from the COVID-19 pandemic experience, many countries could have leveraged innovations better if they had robust and dedicated institutional mechanisms that push for problem-driven innovations, especially for remote communities [37]. It is important to identify and find ways to support these nodes of reform, as they are the poles around which strong and sustainable systems can emerge. The following are some examples of key enablers that a strong health care innovation ecosystem in low-resource settings should fully exploit:

- *High-level national leadership and coordination*: Governments are now starting to play a key role in stewarding and coordinating the innovation ecosystem, although this is not fully in place in most African countries. National governments are key ecosystem actors that can play a key role in regulation; strengthening the linkages between industry, science and academia; creating favorable policy frameworks for innovation; and setting the overall agenda for health innovation based on evidence and priority areas [38]. Ecosystem governance and orchestration of the healthcare innovation ecosystem is still an area that is understudied [39]. This reinforces the call for a study into the roles, particularly, intermediation in ecosystems [40].
- *Technological infrastructural dynamics*: In a study conducted on COVID-19 technological innovations by WHO AFRO, it was found that most responsive health systems relied heavily on the existing technological infrastructures [41]. However, this is a consistent problem in Africa. It is therefore important for African countries to prioritize investment in key infrastructural developments such as ICT backbones and cellphone towers that will enable the use of technologies such as Artificial Intelligence to be utilized in low-cost diagnosis. Additionally, such infrastructure allows for the use of telemedicine in hard-to-reach areas, which can systemically integrate and capacitate the health service delivery process.
- *Funding and innovation financing*: Even though most African governments do have budgetary allocations to support science and innovation, this is not enough to meet the needs of the ecosystem. External, short-term funding dominates the landscape, and this causes a high level of disruption as once funding is finished, some technologies and interventions become obsolete. Additionally, funding mechanisms often focus on individual projects or products and not on understanding or seeking to improve the ecosystem [2]. In 2020, only 16 African countries were ranked in the top 200 countries when it comes to investing in innovation in 2020 [42]. This means that few African countries significantly invest in innovation.
- *Data generation and management disparities*: Absent, incomplete or poor-quality health data undermine the confidence of planners to use data to make decisions. Strong data systems are a vital precondition for health planning, adaptation and innovation [3].
- *Monitoring innovation activities*: Quite a number of countries do not have mechanisms to scan, map or integrate the innovations that are happening in-country or across the region. This creates disparities as more and more solutions that assist with improving service delivery, enabling health promotion and improving health impact usually stem from external sources such as developed countries through donor partners. Measuring the impact from such technologies and innovation ecosystems becomes more difficult to ascertain due to a lack of clear indicators [19].
- *Support local innovations*: The COVID-19 pandemic demonstrated the fragility of healthcare manufacturing supply and value chains. This forced a global recognition of the importance of creating support structures, especially in developing countries, to promote local manufacturing [4]. When it comes to scaling local innovations and strengthening health systems’ capacities, then creating an enabling environment is a fundamental key aspect.

## 6. Conclusions

The Healthcare Innovation Scaling Framework (HISF) is a reference guide that can be utilized by key players both in the health sector and outside to assess the capacity of the local innovation ecosystem that promote sustainable scaling of impactful innovation. The framework highlights key areas that countries should focus on in a bid to strengthen their local innovation systems through creating an enabling environment through which intermediaries or key institutions can support governments in improving national innovation ecosystems. The framework will assist in the following:

- Identifying healthcare innovation ecosystem gaps and needs at the country level;
- Identifying the key strengths and obstacles deterring coordination and alignment of stakeholders within the healthcare ecosystem;
- Designing targeted and tailored recommendations for creating a stronger innovation ecosystem within African countries;
- Mapping out capacity building requirements;
- Undertaking informed resource mobilization efforts that spur and attract collaborative engagement in the ecosystem.

This paper proposes a framework that can be used to assist in scaling innovations by outlining activities that can lead to evidence and data-driven policy. The proposed possible activities can morph to the identification of clearly outlined indicators that can be used as a measurement instrument. Of importance, especially in developing countries, a lot of ecosystem activity is intangible, and hence, this makes measuring the impact of various ecosystems particularly in healthcare a nearly impossible task. This is seen as a foundational framework that is expected to evolve and incorporate other research around innovations (eco)systems so that tools can be created for policy makers and ecosystem builders to be able to assess the different capabilities of their ecosystems being able to support ecosystem-related goals and activities and to promote innovations. The main limitation of this article is that it is based off one dataset, which may not be representative of all innovators across Africa. Looking at other datasets from other innovation ecosystems or across other industries is a proposed next step in building a generic framework that can be utilized across various industries.

## Figures and Tables

**Table 1:** Summary of challenges innovators are facing.

List of Challenges	Percentage of Responses
Lack of knowledge and data sources	17.53%
Insufficient human resource capacity	45.36%
Lack of standardized technology architecture	42.27%
Insufficient technology transfer processes	29.90%
Lack of clear guidance related to intellectual property management	15.46%
Lack of clear guidance on contractual agreements	17.53%
Lack of directives on current trends in the healthcare sector which inform the innovative solutions	20.62%
No prototyping and pilot capabilities	9.28%
Lack of institutional support aligned with developing standards, informing policy and work practices	23.71%
Financial constraints	88.66%
Access to markets	31.96%
Lack of business tools	29.90%
No de-risking mechanisms	7.22%
Other (please specify)	6.19%

**Table 2:** Innovator-identified ecosystem needs.

List of Challenges	Percentage of Responses
Linking to markets for the innovations	65.98%
Capacity building	62.89%
Network building (e.g., through networking or open innovation platforms)	58.76%
Co-creating/Co-designing in the innovation process and technology transfer	42.27%
Innovation management system, i.e., marketing, IP management	29.90%
Brokering—representing actors and negotiating on their behalf or contractual advice	17.53%
Demand articulation, i.e., foresight and forecasting of health needs and requirements	30.93%
R&D, prototyping and piloting capabilities	27.84%
Institutional support—e.g., developing standards, informing policy and work practices	45.36%
Funding	93.81%
Education and training	41.24%
Knowledge sharing	42.27%
Fiscal and nonfiscal incentive mechanisms	19.59%
Well-articulated national innovation agenda	31.96%
Other (please specify)	1.03%

**Table 3:** Framework Indicators.

Category	Component	Possible Indicators [26,35,36]
Policy alignment	Vision, Scope & Goals	Roadmap with key milestones for implementation of strategies
	Institutional capacity	Implementation plan(s) Accountability framework for ethics and human rights Skills matrix (gaps and availability) Innovation register with stakeholders and innovations across interventions
	Continuity and sustainability	Sustainability plan(s)
	Core Interaction	Channels of communication
Stakeholder Management	Public—Private engagement mechanisms	MOUs for engagement with private sector Sustainability plans for engagements
	Regional coordination and compatibility alignment	Policies for regional collaborations Number of memberships and collateral innovation related agreements
	Actor responsibility	Role outline for ecosystem actors
Ecosystem Governance	Supportive fiscal policies, tax reform	Fiscal policies aligned with healthcare innovations Policy follow up
	Investment in relevant skills (education, training)	Leadership and staff aware of innovation processes and concepts Innovation expert pool Innovation resources and tools
	Tensions	Conflict resolution protocols
	Technology Usage	Promote networks around technology Legitimizing technology Promote technology platforms
Knowledge Creation & Diffusion	R&D	Formal agreements with academic institutions
	Support & Services	Expert advisory group on collaboration mechanisms
	Key activity Mapping	Database of innovation ecosystem actor activities
	Tools	Spreadsheets Data analytics (SPSS, R) Business Intelligence tools Geographic information systems (ArcGIS)
	Innovation Activities	Visibility of innovation activities Number of events (e.g., hackathons, webinars, open innovation competitions trainings) done annually
	Education and Training	Courses for upskilling innovation management processes Support for innovators (e.g., with innovation tools such as business model canvas, business plan formulation, market analysis, etc.)
Knowledge Management	Knowledge management Internal Processes	Databases and platforms Accessibility of platforms and knowledge bases
	Risk Management	Stakeholder assessment matrix Data and information recovery plan
	Knowledge management and sharing policies (Trust & Loyalty)	Polices on knowledge management Intellectual property policies
	Strategic Communications	Email bulletins; Portals; Newsletters
Learning culture	Data analytics usage	Data visualization, e.g., dashboards Descriptive analysis Inferential statistics predictive analytics usage
	Ability to Share and Innovate	Data repository availability and accessibility, e.g., Open data
	Value Co-Creation	Collaboration mechanisms in the ecosystem Invest in vehicles and institutions for supporting ideation, proof of concept, incubation, acceleration of innovations
	Accountability	Accountability mechanisms
	Continuous learning	Grassroots curriculum on innovation
Technology Infrastructure	Scalability	Coverage of innovation technologies Mechanisms for scaling
	Interoperability	Interoperability of applications and software
	Feedback Methods	Chatbots, Call centers, SMS functionalities
	Data Privacy and Security	Data and privacy policies
	Data Governance and Storage	Data storage mechanisms and policies in place Centres of power are clear
	Standards	Standards for innovative technologies
Monitoring and Evaluation	Impact assessments (measurement)	Clear Outlined targets and KPIS, e.g., o Health Innovation Index Performance o WHO health innovation index o Health Indicators (e.g., life expectancy, maternal mortality, etc.) Healthcare budgetary spend versus Pan African targets
	Feedback Mechanisms	Annual Surveys Training feedback Event feedback and follow up
Strategic Partnerships	Collaboration mechanisms	Mobilizing cooperation and conducting advocacy activities e.g for resources) Promoting visibility of ecosystem actors Access to Informal communities
	Access to human and physical resources	Human capital: education, specialized training programs Physical resources: natural resources and infrastructure developments
	Financial mechanisms	Financial capital: venture capital, public seed money, financial incentives/loans private investments, financial incentives, grants
Market Shaping	Incentives and mechanisms to promote innovation	Provision of subsidies (share cost of investment) Public procurement support
	Preferential procurement	Regulatory reform supporting niche markets
	Regional and global trade agreements	Specific tax regimes, incentives and exemptions
	Standardization	New standards that improve the environment
	Community engagement	Promote interest and advocacy coalitions

## Data Availability

Not applicable.
